# HBV core promoter mutations and AKT upregulate S-phase kinase-associated protein 2 to promote postoperative hepatocellular carcinoma progression

**DOI:** 10.1038/srep35917

**Published:** 2016-10-25

**Authors:** Lubiao Chen, Lin Gu, Yurong Gu, Hongbo Wang, Meihai Deng, Zania Stamataki, Ye Htun Oo, Yuehua Huang

**Affiliations:** 1 Department of Infectious Diseases, The Third Affiliated Hospital of Sun Yat-sen University, Guangzhou, China; 2 Guangdong Provincial Key Laboratory of Liver Diseases Research, The Third Affiliated Hospital of Sun Yat-sen University, Guangzhou, China; 3 Department of Hepatobiliary Surgery, The Third Affiliated Hospital of Sun Yat-sen University, Guangzhou, China; 4 Institute of Biomedical Research and NIHR Center for Liver Disease, University of Birmingham, Birmingham, United Kingdom

## Abstract

Mutations in the hepatitis B virus (HBV) core promoter (CP) have been shown to be associated with hepatocellular carcinoma (HCC). The CP region overlaps HBV X gene, which activates AKT to regulate hepatocyte survival. However, the cooperation between these two cascades in HCC progression remains poorly understood. Here, we assayed virological factors and AKT expression in liver tissues from 56 HCC patients with better prognoses (BHCC, ≥5-year survival) and 58 with poor prognoses (PHCC, <5-year survival) after partial liver resection. Results showed double mutation A1762T/G1764A (TA) combined with other mutation(s) (TACO) in HBV genome and phosphorylated AKT (pAKT) were more common in PHCC than BHCC. TACO and pAKT levels correlated with proliferation and microvascularization but inversely correlated with apoptosis in HCC samples. These were more pronounced when TACO and pAKT co-expressed. Levels of p21 and p27 were decreased in TACO or pAKT overexpressing HCC due to SKP2 upregulation. Levels of E2F1 and both mRNA and protein of SKP2 were increased in TACO expressing HCC. Levels of 4EBP1/2 decreased and SKP2 mRNA level remained constant in pAKT-overexpressing HCC. Therefore, TACO and AKT are two independent predictors of postoperative survival in HCC. Their co-target, SKP2 may be a diagnostic or therapeutic marker.

Hepatocellular carcinoma (HCC) is one of the most common primary malignancies and the third most frequent cause of cancer-related death worldwide^1^. Risk factors for its development include chronic infection with hepatitis B virus (HBV) or hepatitis C virus, alcohol abuse, aflatoxin exposure and metabolic liver diseases^2^. Despite a successful vaccination program, HBV is predicted to remain the leading aetiological factor of HCC in patients over 25 years of age in coming decades. Worldwide, more than half of all HCC cases occur in China, where almost 100 million people are seropositive for HBV surface antigen (HBsAg)^3^,^4^. Among those individuals with chronic HBV infection, several viral factors, including the HBV genotypes C, Aa and F; a high viral load; and viral mutations have been reported to be associated with a high risk of HCC^5^,^6^,^7^.

The double mutation A1762T/G1764A (TA) in the HBV basal core promoter (CP) region has been widely recognized to be independently associated with HCC^5^,^6^,^8^. In a study of 2762 people with chronic HBV infection who were followed for 13 years, TA was associated with a hazard ratio of 1.73 for HCC development^6^. In addition to TA, other CP mutations, notably C1653T, T1674C/G, C1766T, T1753V and T1768A, have also been reported to be associated with an increased risk of HCC^8^,^9^,^10^,^11^. Moreover, longitudinal studies have shown that TA is selected earlier, whereas other mutations in the CP region gradually accumulate during HBV-induced hepatocarcinogesis^8^,^12^,^13^,^14^. These other mutations are almost invariably associated with TA, suggesting a key role for TA, but combination with additional mutations in the CP region is necessary for HCC development^8^,^12^,^13^. Lastly, HBV mutations are not only related to hepatocarcinogenesis but also HCC survival^15^,^16^.

Although HCC has been studied for several decades, current treatment for this disease is limited and generally ineffective, and the 5-year survival rate for all disease stages combined is less than 5%^17^,^18^. At present, tumour resection or liver transplantation is the only potentially curative surgical treatment. Indeed, survival among patients with HCC is relatively poor, even in patients who undergo surgical resection, and the recurrence rate is high. Nevertheless, a better understanding of the mechanisms of HCC progression may lead to the identification of potential diagnostic markers or therapeutic targets.

AKT is the most frequently activated oncoprotein in human cancers, including HCC. Specifically, the hyperactivation of AKT (phosphorylated AKT, pAKT) contributes to carcinogenesis via multiple mechanisms, such as increased cell proliferation, the inhibition of apoptosis, and the abrogation of cell cycle progression checkpoints^19^,^20^. Moreover, the overexpression of AKT has been observed in human HCC samples, and AKT may phosphorylate many substrates that are conducive to carcinogenesis and tumour progression^21^. However, the most critical targets for AKT-mediated cell proliferation and oncogenic transformation in HCC have not yet been identified. We previously showed that S-phase kinase-associated protein 2 (SKP2), the most critical downstream effector of AKT, is upregulated by mutations in the HBV CP region, which consequently downregulates p21 to accelerate cell cycle progression, anchorage-independent cell growth, and cellular proliferation^22^. Accordingly, SKP2 plays an important role in HBV CP mutation-induced carcinogenesis.

Despite the importance of AKT and mutations in the HBV CP region in hepatocarcinogenesis, their functional interaction has not yet been linked to prognosis after the surgical removal of cancer. Here, we hypothesized that AKT and HBV CP mutations cooperate to disrupt SKP2, which accelerates tumour proliferation and angiogenesis while inhibiting tumour apoptosis. This mechanism ultimately results in unrestrained HCC growth and consequently shortens survival. In this study, we examined the HBV genome in liver tissue and, based on the expression in the CP region, classified these samples as either wild-type (WT) samples, TA-expressing samples or samples that expressed TA and at least one other mutation (TACO). Specifically, these other mutations were A1768, A/C/G1753, T1766 and T1653. The effect of AKT and HBV CP mutation co-expression on prognosis was evaluated. Finally, our findings imply that concurrent high levels of pAKT and TACO expression may serve as a valuable prognostic marker in patients after surgical resection. Consequently, SKP2 may be a potential therapeutic target in HBV-associated HCC.

## Results

### Patient characteristics

A total of 139 patients with HBV-related HCC were enrolled after liver resection and followed up for 5 years. Nineteen patients were lost, and another 6 were negative for HBV DNA; the remaining 114 patients were further analysed in the study. All patients were Asian, and almost 85.1% of the cohort was male. Most patients had a background of cirrhosis, and the mean age was 46.1 ± 11.0 years with a mean survival length of 46.6 ± 18 months. The majority (86.0%) of patients had a single tumour, and the mean size of the largest tumour was 7.43 ± 4.50 cm. Notably, the presence of cirrhosis, a larger tumour, multiple lesions, a higher AST, and lower albumin levels were factors that significantly associated with shorter HCC survival. Conversely, age, sex, encapsulation, the TNM classification, the CHILD classification, the AFP level, the ALT level, the bilirubin level, the prothrombin time, the creatinine level, the presence of portal vein thrombosis, and alcohol consumption were not significantly associated with survival (Supplementary Table S1).

### Concomitant expression of AKT and HBV CP mutations is associated with HCC prognosis

**Figure 1:**
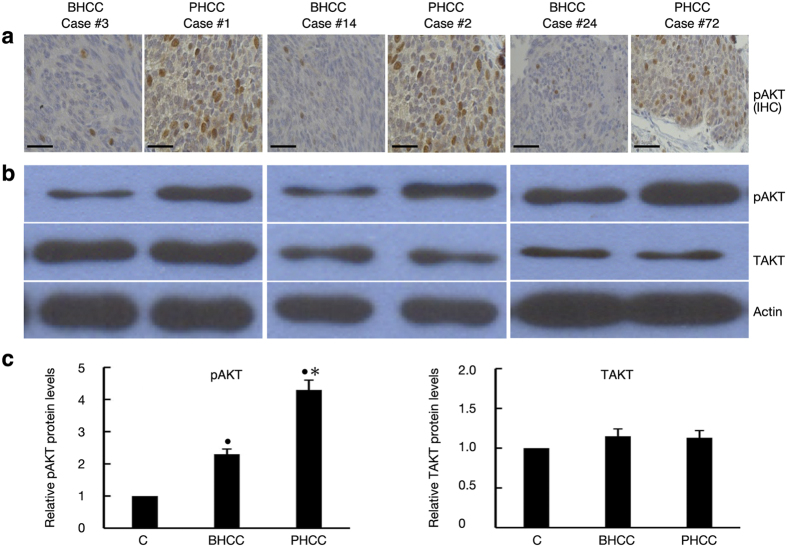
Expression of pAKT in human HCC. (**a**) pAKT expression was assessed by immunohistochemistry in liver tissues from patients with a better or poor prognosis. Representative images are shown for three patients in each category (Scale bar, 50 μm). (**b**) Western blotting confirmed that the protein expression of pAKT corroborated the immunohistochemical analyses of these 6 representative cases. Total AKT (TAKT) and actin levels were used as protein loading controls. (**c**) Relative protein expressions of pAKT and TAKT quantified by densitometry in livers of 56 BHCC and 58 PHCC, respectively. Bands were relative to protein expression from surrounding non-HCC tissues (C, control, normalized as 1). ^•^*P* < 0.05 compared with control; ^*^P < 0.05: PHCC vs. BHCC.

Of all 114 patients, 56 patients had a better prognosis (BHCC) and 58 patients had a poor (PHCC) prognosis. BHCC and PHCC were defined as survival ≥5 years and survival <5 years, respectively. Moreover, pAKT1 expression was more common in the PHCC population than in the BHCC population (55.2% vs. 32.1%; *P* = 0.015) (Table 1). Moreover, both immunohistochemistry (IHC) and western blot analyses of HCC specimens indicated that pAKT1 was overexpressed in patients exhibiting shorter survival, although similar levels of total AKT were found in both HCC subgroups (Fig. 1).

A virologic analysis of CP mutations from HBV DNA extracted from noncancerous liver tissue showed that patients in the PHCC group developed more mutations than patients in the BHCC group. Furthermore, TACO mutations were more common in the PHCC group than in the BHCC group (72.4% vs. 46.4%; *P* = 0.007). In contrast, the wild-type CP region was less common among PHCC patients than BHCC patients (10.3% vs. 28.6%; *P* = 0.018), whereas the TA expression rates (in the absence of other mutations) were similar between the two groups (17.2% vs. 25.0%; *P* = 0.362) (Table 1).

A Cox regression analysis was used to examine the association between clinicopathological or virologic factors and overall survival after surgical resection in HBV-related HCC. Of all patients included, the cause of death was documented in 19 patients. Liver failure with hepatoencephalopathy and subsequent multi-organ failure occurred in 5 patients; progressively enlarged HCC with tumor rupture and shock occurred in 8 patients; lung metastasis and respiratory failure developed in 5 patients; esophageal varices bleeding with hypovolemic shock occurred in 1 patient. According to a univariate analysis, a tumour size >5 cm (hazard ratio [HR] = 3.083, 95% confidence interval [CI] = 1.008–9.434, *P* = 0.048), HBV DNA > 6.0E+06 copies/ml (HR = 4.283, 95% CI = 1.240–14.796, *P* = 0.021), TACO mutations (HR = 2.815, 95% CI = 1.010–7.846, *P* = 0.048), a higher HBsAg titre (HR = 3.987, 95% CI = 1.237–10.712, *P* = 0.021), and strong pAKT1 expression (HR = 3.896, 95% CI = 1.308–11.605, *P* = 0.01) were associated with significantly increased mortality due to HCC. After adjusting for other confounding factors, a multivariate analysis showed that HBV DNA > 6.0E+06 copies/ml (HR = 4.496, 95% CI = 1.218–15.116, *P* = 0.012), TACO mutations expression (HR = 3.007, 95% CI = 1.075–8.745, *P* = 0.029), and strong pAKT1 expression (HR = 4.022, 95% CI = 1.037–12.942, *P* = 0.039) were significantly associated with a shorter overall survival (Table 2).

**Figure 2:**
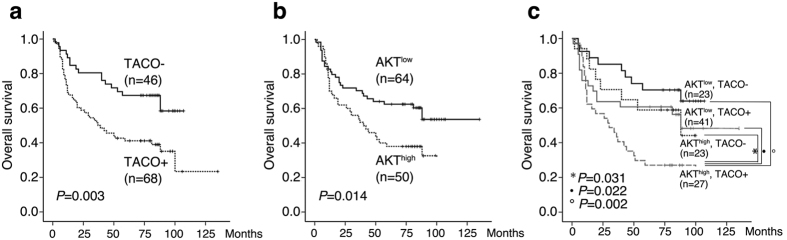
Association between postoperative survival, intrahepatic HBV CP mutations and pAKT levels. (**a**) Comparison of survival between patients with and without TACO mutations. (**b**) Comparison of survival between patients with different levels of pAKT. (**c**) Combination of intrahepatic TACO mutations and pAKT as a predictor for overall survival. TACO−: Negative for TACO; TACO+: Positive for TACO; AKT^low^ and AKT^high^: low or high levels of pAKT.

Because strong pAKT1 expression and presence of TACO are important independent predictors of survival, a Kaplan-Meier survival analysis was performed to assess the predictive values of high pAKT1 expression, TACO mutations, or the co-expression of both these factors. As expected, the cumulative survival rate calculated based on TACO expression suggested that CP mutations were predictive of overall survival in patients with HBV-related HCC (*P* = 0.003) (Fig. 2a). Likewise, the pAKT1 levels also significantly predicted survival among patients with HBV-related HCC (*P* = 0.014) (Fig. 2b). When these 2 factors were combined, 23 (20.2%) patients expressed low levels of pAKT1 without a TACO mutation, 41 (36.0%) patients expressed low pAKT1 levels with a TACO mutation, 23 (20.2%) patients expressed high pAKT1 levels without a TACO mutation, and 27 (23.6%) patients expressed high pAKT levels and a TACO mutation. Importantly, postoperative survival was shortest among patients expressing high levels of pAKT and a TACO HBV mutation (Fig. 2c).

Similarly, the Cox proportional hazard model was used to examine the association between clinicopathological and virological factors and disease-free survival after surgical resection of HBV-related HCC. Of the 114 HCC patients, 34 did not have positive results while 80 experienced tumor recurrence or metastasis during the follow up period. Multivariate analysis of factors associated with tumor progression indicated that ascites, prothrombin time>12 seconds, AST>ULN, portal vein thrombosis, AFP>400 ng/ml, and positive TACO were associated with a shorter disease-free survival (DFS) (Supplementary Table S2). Higher proportion of patients without TACO had better DSF than patients with TACO infection (19.1% vs. 45.7%, *P* = 0.003). When we stratified patients in subgroups of either presence or absence of TACO mutations, higher proportion of patients with high AKT level than those with low AKT level were found to have shorter DFS (28% vs. 61%, *P* = 0.037) in TACO negative group. However, comparable results of DFS were observed among patients in TACO positive group regardless AKT level (19.5% vs. 22.7% for patients with low or high AKT level, respectively. *P* = 0.589). Kaplan-Meier survival analysis also showed different prediction power of TACO and AKT regarding to tumor free survival in current HBV-related HCC cohort (Supplementary Figure S1a–c).

Overall, these data indicate that the combination of AKT expression and TACO mutations is associated with accelerated tumour progression in HBV-related HCC.

### Additive effects of AKT1 and TACO mutations on cell proliferation and sustained human HCC growth

**Figure 3:**
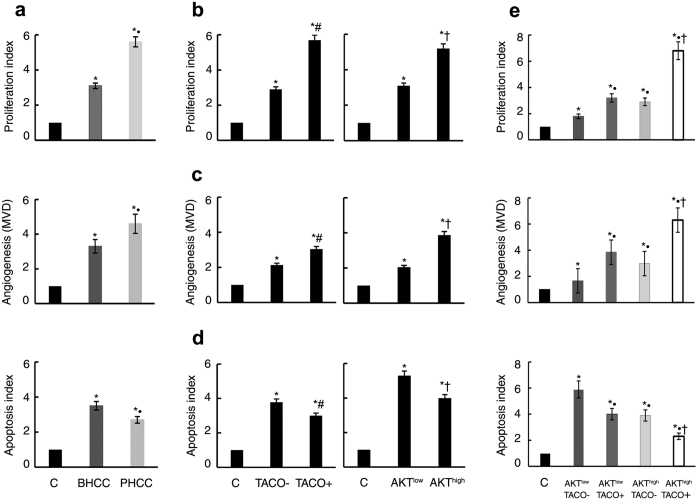
Associations of AKT levels and TACO expression with cell proliferation, apoptosis and the angiogenesis index in human HCC. (**a**) Proliferation, angiogenesis, and apoptosis indices in HCC subgroups. Bars indicate values relative to paracancerous tissues. The data represent the mean ± SD of 56 patients in the BHCC group and 58 patients in the PHCC group. ^*^P < 0.05 compared with control, ^•^*P* < 0.05: PHCC vs. BHCC. Indices of proliferation (**b**), MVD (**c**), and apoptosis (**d**) in HCCs harbouring CP WT HBV or TACO mutant HBV (*left*) and in HCCs expressing different levels of pAKT (*right*). ^*^P < 0.05 compared with control; ^#^*P* < 0.05: HCC with TACO+ vs. HCC with TACO−; ^†^*P* < 0.05: HCC with AKT^high^ vs. HCC with AKT^low^. (**e**) Above indices for AKT^high^ and TACO+ combined expression. ^*^P < 0.05 compared with control; ^•^*P* < 0.05: HCC with AKT^low^/TACO+ or AKT^high^/TACO− or AKT^high^/TACO+ vs AKT^low^/TACO−; ^†^*P* < 0.05: HCC with AKT^high^/TACO+ vs. AKT^low^/TACO+ or AKT^high^/TACO− or AKT^low^/TACO−. All controls were normalized as 1.

To explore the cellular mechanisms responsible for accelerated HBV-related HCC progression, we evaluated the proliferation, apoptosis, and angiogenesis indices in this HCC cohort (All controls were normalized as 1). Compared with tissues from BHCC patients, tissues from patients with PHCC displayed higher indices of proliferation (5.6 vs. 3.1, *P* = 0.043,) and angiogenesis (4.6 vs. 3.1, *P* = 0.031) and a lower index of apoptosis (2.7 vs. 3.5, *P* = 0.046) (Fig. 3a). Similarly, HCC samples expressing high levels of pAKT contained more Ki67-positive cells than samples expressing low levels of pAKT (5.2 vs. 3.1, *P* = 0.014). The proliferation index was also significantly higher in TACO positive tumours than in tumours not infected with a TACO mutant HBV (5.7 vs. 2.9, *P* = 0.034) (Fig. 3b). Patients with high pAKT expression or infection with TACO mutant HBV exhibited 1.6- (*P* = 0.002) and 1.9-fold higher (*P* = 0.001) microvessel density (MVD) than patients with low pAKT levels or patients who did not harbour a TACO mutant HBV infection, respectively (Fig. 3c). However, the apoptotic index inversely correlated with the levels of pAKT and TACO mutations in human HCC (3.0 vs. 3.8 for high pAKT and low pAKT tumours, respectively, *P* = 0.046; and 4.0 vs. 5.3 for TACO positive and TACO negative tumours, respectively, *P* = 0.041) (Fig. 3d). Notably, the index of cell proliferation was maximized when high levels of pAKT and a TACO mutation were co-expressed [6.8 vs. 3.2 (*P* = 0.028) or 2.9 (*P* = 0.006) for combined high pAKT and TACO vs. TACO or high pAKT alone, respectively]. Furthermore, the microvessel density was maximized [6.3 vs. 3.8 (*P* = 0.013) or 2.9 (*P* = 0.041) for combined high pAKT and TACO vs. TACO or high pAKT alone, respectively] and apoptosis was minimized [2.3 vs. 4.0 (*P* = 0.013) or 4.1 (*P* = 0.041) for TACO or high pAKT alone, respectively] in these samples (Fig. 3e). Taken together, these results suggest that the pAKT levels and TACO mutation status correlate with increased proliferation, angiogenesis index and apoptosis in HCC.

### Co-expression of AKT and TACO correlates with the levels of the cell cycle oncosuppressors p21 and p27

**Figure 4:**
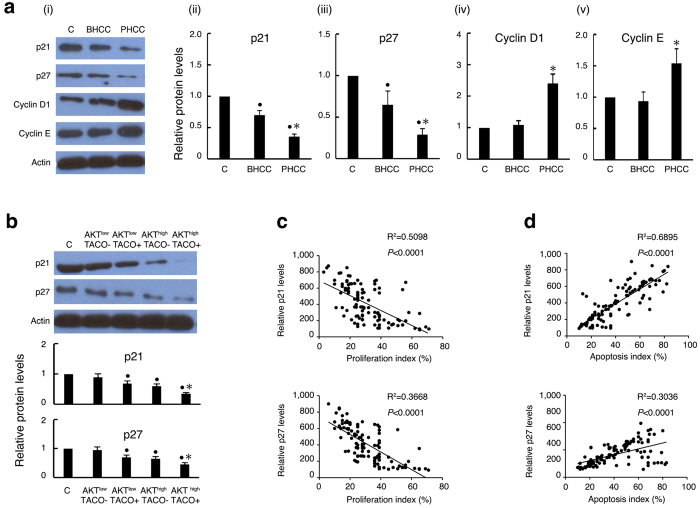
Correlation of pAKT and TACO with cell cycle regulators and cell viability in human HCC. (**a**) Expression of cell cycle regulatory genes in human HCC tissues. Representative immunoblotting of cell cycle regulators (i). Relative protein expressions of p21(ii), p27 (iii), Cyclin D1 (iv), and Cyclin E (v), and levels in their surrounding non-HCC livers as control (normalized as 1). ^•^*P* < 0.05 compared with control; ^*^P < 0.05: PHCC vs. BHCC. (**b**) Representative immunoblotting of p21 and p27 in different HCC subtypes (*Upper*), and their relative protein levels (*Lower*). ^•^*P* < 0.05: compared with control; ^*^P < 0.05: HCC with TACO+/AKT^high^ vs. TACO−/AKT^low^ or TACO+/AKT^low^ or TACO−/AKT^high^. Correlation of p21 and p27 with the proliferation index (**c**) and apoptosis index (**d**). The proliferation and apoptotic indices of HCC were measured based on the number of Ki67-positive and apoptotic cells, respectively, and are expressed as a percentage of total hepatocytes.

We and others showed that cell cycle regulators affect hepatocarcinogenesis and tumour progression by modulating cellular growth and apoptosis^22^,^23^. Consequently, we measured the expression of cell cycle inhibitors and stimulators in our HCC cohort to further substantiate the probable mechanisms by which AKT1 or TACO mutations affect cell growth. Specifically, p21 and p27 have been identified as important checkpoint regulators for the G1-S and G2 transitions and the progression of the cell cycle, and they are oncosuppressors. Moreover, p21 and p27 inhibition favours the uncontrolled progression of injured or even tumour cells^24^,^25^. Therefore, we first compared the levels of p21 and p27 in different HCC subgroups and found that the levels of p21 and p27 were decreased, whereas those of cyclin D1 and cyclin E were increased in the PHCC cohort (Fig. 4a). We then assessed the role of AKT, TACO mutations or the combination of these two factors on cell cycle inhibitors. As expected, the p21 and p27 levels were reduced in HCC samples expressing high levels of pAKT or a TACO mutation compared with samples in which AKT expression was low and samples harbouring wild-type HBV infections. Moreover, these cell cycle regulators were significantly downregulated in tissues simultaneously expressing high levels of pAKT and TACO mutation (Fig. 4b). These data indicate that the p21 and p27 expression levels correlate with the pAKT level or TACO mutation status and corroborate the differences observed between the BHCC and PHCC subgroups. Moreover, the levels of p21 and p27 trended inversely with proliferation (Fig. 4c) and directly correlated with apoptosis (Fig. 4d). Overall, our results show that the pAKT levels and TACO expression predicted a poor HCC prognosis, and reduced expression levels of cell cycle regulators are linked to proliferation and apoptosis.

### SKP2 levels correlate with poor prognosis in HBV-related HCC

**Figure 5:**
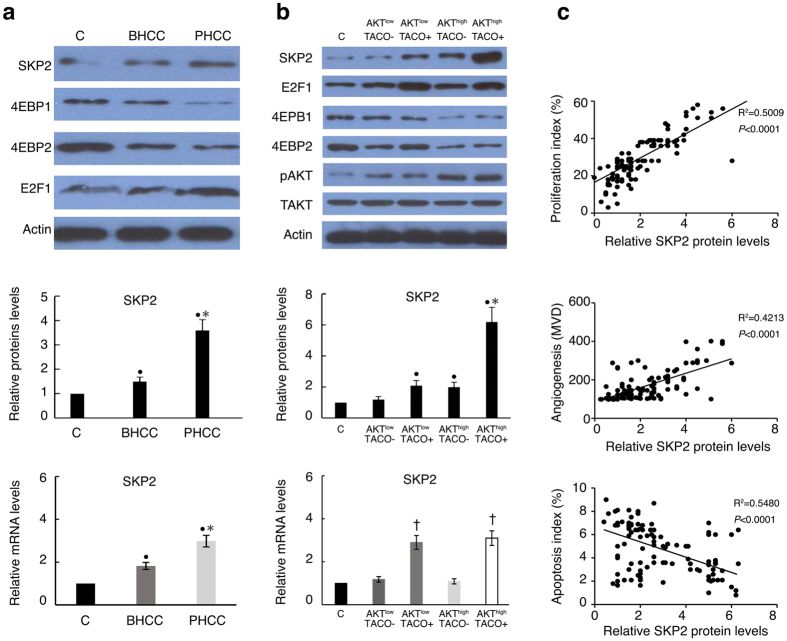
Effect of AKT and TACO on the levels of SKP2 via independent pathways. (**a**) Representative Western blot analysis of SKP2, transcription factor E2F1 and translation inhibitor 4EBP1/2 (*upper*), relative protein (*middle*) and mRNA levels (*lower*) of SKP2, in the BHCC and PHCC subgroups as well as paracancerous tissues, which served as a control. ^•^*P* < 0.05: compared with control. ^*^P < 0.05: PHCC vs. BHCC. (**b**) Above protein expressing by Western blot analysis (*upper*), relative protein (*middle*) and mRNA levels (*lower*) in HCC with different levels of AKT expression and HBV CP status. ^•^*P* < 0.05: compared with control. ^*^P < 0.05: HCC with TACO+/AKT^high^ vs. TACO−/AKT^low^ or TACO+/AKT^low^ or TACO−/AKT^high^. ^†^*P* < 0.05: HCC with TACO+ vs. TACO− or control. (**c**) Correlation of SKP2 protein levels with proliferation index, angiogenesis (MVD), and apoptosis index in 114 human HBV-associated HCC cases. Protein expression quantified by densitometry was shown as a relative value compared to the control. All controls were normalized as 1.

SKP2 has been identified as a molecule downstream of AKT^26^ and can target p21 and p27 for proteasomal degradation^27^. We previously showed that HBV CP mutations increase SKP2 expression and are thus involved in hepatocarcinogenesis^22^. Here, progressive increases in both the mRNA and protein levels of SKP2 were found from BHCC to PHCC (Fig. 5a) and from HCC with lower levels of pAKT and negative TACO to combined strong pAKT and positive TACO (Fig. 5b). Moreover, the SKP2 level directly correlated with the proliferation index (R^2^ = 0.5009, *P* < 0.0001) and MVD (R^2^ = 0.4213, *P* < 0.0001) and inversely correlated with apoptosis (R^2^ = 0.5480, *P* < 0.0001) (Fig. 5c). As expected, SKP2 protein expression was higher in HCC samples that overexpressed AKT and TACO compared with samples that expressed low levels of AKT and samples harbouring a wild-type CP HBV infection, respectively. Accordingly, SKP2 expression was maximized in HCC samples co-expressing AKT and a TACO mutation. These results suggested that SKP2 is likely a key factor in the mechanism that leads to pAKT upregulation in HCC, particularly in TACO HBV-associated HCC progression.

Surprisingly, the increased level of SKP2 mRNA is likely related only to TACO expression and does not depend on the level of pAKT (Fig. 5b). Indeed, SKP2 mRNA expression was similar in HCC samples expressing low and high levels of pAKT, suggesting that the SKP2 mRNA and protein levels do not correlate and that AKT may mediate SKP2 expression by affecting translation or degradation.

AKT has been shown to modulate mRNA translation primarily via mTORC1, which depends on the downstream effector eIF4E^28^. Specifically, a translation factor named 4E-binding protein (4EBP) binds to eIF4E to impede mRNA translation^26^. As shown in Fig. 5 and Supplementary Figure S2, the levels of 4EBP1 and 4EBP2 were decreased in HCC samples expressing high levels of pAKT and in the PHCC subgroup. This inhibition of 4EBP may lead to eIF4E hyperactivation, which promotes SKP2 translation. Conversely, mutations in the HBV CP region have been found to mediate SKP2 transcription via E2F1^29^. Accordingly, the levels of E2F1 were significantly increased in TACO expressing HCC tissue (Fig. 5b, Supplementary Fig. S2c), suggesting that HBV CP mutations may induce hepatocarcinogenesis and tumour progression via similar mechanisms.

## Discussion

Increasing evidence indicates that the aberrant activation of AKT1 is a key oncogenic event in human hepatocarcinogenesis^30^. Specifically, cell- and animal-based experiments show that AKT1 interacts with several HBV proteins to contribute to deterioration in liver disease^31^. Moreover, some studies illustrate that HBV mutations are closely associated with HCC development and tumour progression^15^,^16^. Although some growth regulators have been demonstrated to be good prognostic tools in many other common cancers, the connection between HBV CP mutations and AKT1 has not been carefully investigated by examining the prognostic value of HBV-associated HCC. Here, we assayed both virological factors and AKT1 activation in liver tissues obtained from HCC patients after tumour resection. Our data demonstrate that the co-expression of activated AKT and TA-combined mutations (TACO) affected postoperative prognosis in human HCC, providing strong genetic evidence that the combination of pAKT and TACO plays a pivotal role in HCC progression.

We found that pAKT levels were higher and TACO mutations were more common in the PHCC subgroup than in the BHCC subgroup, suggesting that the additive effects of pAKT and TACO may play a specific role in the outcome of HBV-associated HCC. Because rapid malignant transformation and tumour progression result in an unfavourable prognosis for patients with cancer, and because pAKT and/or TACO may accelerate HCC tumour cell growth, these molecules may be therapeutic targets. Indeed, our results confirmed that high pAKT levels together with TACO expression resulted in HCC progression by increasing cell proliferation and angiogenesis and conferring anti-apoptotic advantages to the liver lesion. This finding is consistent with the fact that AKT overexpression and TA mutations promote hepatocarcinogenesis and tumour progression in AKT transgenic mice and human HCC samples, respectively^15^,^17^. These observations, together with the observed aberrant Ki67 expression, MVD, and apoptotic indices in PHCC, support the hypothesis that the stabilization of pAKT and the accumulation of mutations in the CP region of the HBV genome have a potential prognostic role in HBV-associated HCC.

Moreover, we found that impairment in cell cycle regulators influenced the overall postoperative survival of our HCC cohort. The decreased levels of cell cycle inhibitors and concurrently higher levels of pAKT and TACO expression in the PHCC subgroup implicate p21 and p27 in the growth and progression of human liver lesions. This behaviour can be attributed to enhanced proteasomal degradation because higher SKP2 levels were accompanied by high levels of pAKT and TACO expression in the PHCC subgroup. Other groups and our group observed that SKP2 levels were directly correlated with the proliferation and MVD of HCC and inversely correlated with the apoptosis index and survival. Accordingly, forced SKP2 expression in human HCC SKI cells triggered a strong increase in proliferation and a decline in the expression of cell cycle regulators^23^.

The HBV CP region overlaps with the coding sequence for the C-terminus in the HBV X (HBx) gene^32^. The HBx protein is an important oncoprotein in HCC pathogenesis^33^, and mutations in this region may alter its biological functions, thereby increasing its oncogenicity. Moreover, pAKT levels and TACO may exert synergistic instead of additive effects on HCC survival because the HBx protein can activate AKT to regulate hepatocyte survival^31^. However, this synergistic effect does not seem to apply to our study because we found a discrepancy in the levels of SKP2 mRNA produced by HCC expressing high levels of pAKT and TACO. The decreases in both 4EBP1 and 4EBP2 in the pAKT-overexpressing HCC samples examined upregulate eIF4E to facilitate SKP2 translation and finally increase its protein level. Furthermore, the simultaneously increased mRNA and protein levels of SKP2 in TACO expressing HCC indicated that SKP2 is transcriptionally regulated because HBV CP mutations target E2F1, a major transcription factor involved in the upregulation of SKP2^29^. Furthermore, the HBV CP mutation status did not significantly affect the levels of pAKT in human HCC (*P* = 0.186, Table 3).

Compared with previous studies that have analysed the impact of HBV on HCC prognosis, our findings are significant for the following reasons. First, previous studies have only analysed virological factors or oncogenes associated with HCC progression, whereas our study showed that the concomitant expression of AKT1 and HBV CP mutations corresponding to the CP mutations that are clinically associated with HCC promote tumour progression. Second, many clinical studies have evaluated factors that are related to HCC prognosis based on 3-year survival, whereas we demonstrated that HBV factors and AKT1 exert an additive effect on the 5-year survival of patients with HCC. Third, we showed, for the first time, that both HBV CP mutations and AKT1 expression increased cell proliferation and angiogenesis, likely via their co-target SKP2.

One limitation of this study is the absence of *in vitro* experiments to corroborate the contribution of pAKT and TACO to HCC progression via the dysregulation of SKP2. However, *in vitro* and *ex vivo* studies have demonstrated that abolishing AKT can increase 4EBP1/2 expression to enhance their binding to eIF4E, which consequently inhibits 5′-cap-dependent mRNA translation. Conversely, overexpressing AKT alleviates the inhibition of mRNA translation by 4EBP1/2 by facilitating 4EBP1/2 dissociation from eIF4E. This effect may enable eIF4E to interact with elf4G to initiate SKP2 mRNA translation, which upregulates SKP2 protein expression to accelerate HCC cell proliferation, transformation, and oncogenesis^17^,^28^. Moreover, our previous studies have shown that TA combined with other CP mutations in the HBV genome enhanced cell cycle progression, cellular growth and colony formation in both HCC cell lines and human primary hepatocytes by influencing SKP2 promoter activity^22^,^29^. Nevertheless, we plan to further validate the current findings in an animal model of disease. Although our observations suggested that the combination of pAKT and TACO affects the prognosis of HBV-associated HCC, other oncogenic processes, such as WNT signalling or HBV replication, may also be associated with HCC progression.

In summary, we discovered that TA combined with other CP mutations and aberrantly high AKT activation might be two independent promising diagnostic markers of postoperative survival among HCC patients. They jointly result in the uncontrolled upregulation of SKP2, which may favour cell transformation and tumour progression. Future work should focus on the development of small molecules that inhibit SKP2, which may delay the progression of more aggressive HCC and improve clinical outcomes.

## Materials and Methods

### Patients and samples

Consecutive HCC patients who underwent total liver tumour resections between January 2004 and March 2007 at the Third Affiliated Hospital of Sun Yat-sen University and who had a history of chronic hepatitis B infection and were currently positive for HBsAg were included. Patients co-infected with immunodeficiency virus (HIV), hepatitis C virus, or hepatitis D virus were excluded. All patients were followed up until May 2012, and their medical records and clinicopathological data were retrospectively reviewed. Overall survival was defined as the interval between the date of surgery and date of last follow-up or HCC-related death, whichever occurred first. After surgical resection, the matched paired tumour and corresponding surrounding non-tumourous liver tissue samples were immediately frozen at −80 °C, and serum samples were stored at −30 °C (details are provided in the supplementary materials).

All experimental protocols in this project were approved by the Ethical Committee of the Third Affiliated Hospital of the Sun Yat-sen University (EC-TAH-SYSU [2013]−2–43). The methods were performed in accordance with the approved guidelines. Written informed consent was obtained from all patients in this study.

### Virological assay

HBV DNA was extracted from noncancerous liver tissues adjacent to the tumour as previously described^15^. The HBV DNA level in the liver tissue was measured using the ABI 7300 TaqMan platform (Life Technologies, Carlsbad, CA). The DNA sequences were aligned using Seqman II and EdiSeq software (DNASTAR Inc., Madison, WI) and compared to consensus sequences of the respective HBV genotype. The mutations TA, A1768, A/C/G1753, T1766, and T1653 in the CP region were verified by direct sequencing.

### Proliferation and apoptotic indices

Cell proliferation in tissues was assayed by immunoperoxidase staining with anti-Ki67 antibody. The proliferation index was determined by counting the number of positive cells per high-power field (HPF, 400× magnification) per 3000 hepatocytes. The apoptotic indices were measured by staining figures with the ApoTag peroxidase *in situ* apoptosis kit (Millipore, Billerica, MA) using 3000 hepatocytes.

### Definition of pAKT levels

All specimens stained for pAKT were scored by 2 independent pathologists according to the proportion and intensity of positive cells within 10 microscopic visual field per slide (200-fold magnification)^34^. Briefly, a proportion score, which represented the estimated proportion of positively stained tumor cells, was classified: <10% as 0; 10–25% as 1; 26–50% as 2; 51–75% as 3; and >75% as 4. An intensive score, which represented the average intensity of the positive tumor cells, was defined: no staining as 0; weak as 1; moderate as 2; strong as 3. The proportion and intensity scores were then multiplied to obtain a total score, and the cutoff point was used to analyze the pAKT value in specimens. Within range of 0–12, cut point ≤ 3 or >3 indicate low and high gene expression, respectively.

### Antibodies, western blotting and immunohistochemistry

The experimental protocol and additional details are provided in the supplementary materials.

### Statistical analysis

Continuous variables are expressed as the mean ± standard deviation. Categorical variables are expressed as numbers and percentages. Continuous variables were compared with a 2-sided Student’s *t*-test or a Mann-Whitney *U*-test depending on the distribution. Categorical variables were compared with a chi-square test or Fisher’s exact test. A Cox regression analysis was used to identify clinicopathological and virological factors associated with postoperative survival. The Kaplan-Meier method was used to estimate the cumulative survival probability. The log-rank method was used to compare survival curves between groups. *P* values less than 0.05 were considered to indicate significant differences, and the data were analysed using SPSS software, version 15 (SPSS Inc. Chicago, IL).

## Additional Information

**How to cite this article**: Chen, L. *et al*. HBV core promoter mutations and AKT upregulate S-phase kinase-associated protein 2 to promote postoperative hepatocellular carcinoma progression. *Sci. Rep*. **6**, 35917; doi: 10.1038/srep35917 (2016).

## Supplementary Material

Supplementary Information

## Figures and Tables

**Table 1:** Both HBV CP mutations and AKT were associated with poor HCC prognosis.

Variables	All patients (n = 114)	BHCC (n = 56)	PHCC (n = 58)	*P* value
Age (years)				
>50	43 (37.7)	19 (33.9)	24 (41.4)	0.445
≤50	71 (62.3)	37 (66.1)	34 (58.6)	
Male, n (%)	97 (85.1)	46 (82.1)	51 (87.9)	0.380
Cirrhosis, n (%)				
Yes	83 (72.8)	35 (62.5)	48 (82.8)	0.020
No	31 (27.2)	21 (37.5)	10 (17.2)	
Tumor size				
>5 cm, n (%)	73 (64.0)	30 (53.6)	43 (74.1)	0.048
≤5 cm, n (%)	41 (36.0)	26 (46.4)	15 (25.9)	
Tumor quantity, n (%)				
≥3	11 (9.6)	1 (1.8)	10 (17.2)	0.001
= 2	5 (4.4)	3 (5.3)	2 (3.5)	0.676
= 1	98 (86.0)	52 (92.9)	46 (79.3)	0.057
Encapsulation, n (%)				
No	40 (35.1)	17 (30.4)	23 (39.7)	0.331
Yes	74 (64.9)	39 (69.6)	35 (60.3)	
CHILD Classification, n (%)				
B	9 (7.9)	4 (7.1)	5 (8.6)	1.000
A	105 (92.1)	52 (92.9)	53 (91.4)	
TNM classification, n (%)				
I	55 (48.2)	28 (50.0)	27 (46.6)	1.000
II+III	59 (51.8)	28 (50.0)	31 (53.4)	
AFP (ng/ml), n (%)				
>400	47 (41.2)	22 (39.3)	25 (43.1)	0.707
≤400	67 (58.8)	34 (60.7)	33 (56.9)	
Ascites, n (%)				
Yes	11 (9.6)	5 (8.9)	6 (10.3)	0.525
No	103 (90.4)	51 (91.1)	52 (89.7)	
Portal vein thrombosis, n (%)				
Positive	5 (4.4)	1 (1.8)	4 (6.9)	0.364
Negative	109 (95.6)	55 (98.2)	54 (93.1)	
ALT (U/L), n (%)				
>ULN	70 (61.4)	34 (60.7)	36 (62.1)	0.711
≤ULN	44 (38.6)	22 (39.3)	22 (37.9)	
AST (U/L), n (%)				
>ULN	41 (36.0)	13 (23.2)	28 (48.3)	0.006
≤ULN	73 (64.0)	43 (76.8)	30 (51.7)	
Albumin (g/L), n (%)				
≥40	80 (70.2)	45(80.4)	35 (60.3)	0.025
<40	34 (29.8)	11 (19.6)	23 (39.7)	
Bilirubin (μmol/L), n (%)				
≥23.9	13 (11.4)	6 (10.7)	7 (12.1)	1.000
<23.9	101 (88.6)	50 (89.3)	51 (87.9)	
Prothrombin time (s), n (%)				
≥12	12 (10.5)	5 (8.9)	7 (12.1)	1.000
<12	102 (89.5)	51 (91.1)	51 (87.9)	
PLT (10E9/L), n (%)				
<170	41 (36.0)	20 (35.7)	21 (36.2)	1.000
≥170	73 (64.0)	36 (64.3)	37 (63.8)	
Alcohol use, n (%)				
Yes	41 (36.0)	19 (33.9)	22 (37.9)	0.699
No	73 (60.0)	37 (66.1)	36 (62.1)	
HBV DNA (copies/ml), n (%)				
>6.0E+06	33 (28.9)	10 (19.6)	23 (37.9)	0.021
≤6.0E+06	81 (71.1)	46 (80.4)	35 (62.1)	
HBsAg (IU/ml), n (%)				
≤500	60 (52.6)	36 (64.3)	24 (41.4)	0.025
>500	54 (47.4)	20 (35.7)	34 (58.6)	
HBV genotypes, n (%)				
B	66 (57.9)	32 (57.1)	34 (58.6)	1.000
C	48 (42.1)	24 (42.9)	24 (41.4)	
CP status, n (%)				
WT	22 (19.3)	16 (28.6)	6 (10.3)	0.018
TA	24 (21.1)	14 (25.0)	10 (17.2)	0.362
TACO	68 (59.6)	26 (46.4)	42 (72.4)	0.007
pAKT1 expression, n (%)				
High	50 (43.9)	18 (32.1)	32 (55.2)	0.015
Low	64 (56.1)	38 (67.9)	26 (44.8)	

Samples in the PHCC group expressed significantly higher levels of pAKT than samples in the BHCC group (55.2% vs. 32.1%; *P* = 0.015). Moreover, TACO mutations were more common in the PHCC group than in the BHCC group (72.4% vs. 46.4%; *P* = 0.007). The data were analysed using SPSS, version 15, and the results are expressed as a percentage. Categorical variables were compared with a chi-square test or Fisher’s exact test. *P* values < 0.05 were considered to indicate significant differences. PHCC, HCC patients with a poor prognosis (less than 5-year survival); BHCC, HCC patients with a better prognosis (more than 5-year survival); ALT, alanine transferase; AST, aspartate transferase; AFP, alpha fetal protein; PLT, platelet; ULN, upper limit of normal; LLN, lower limit of normal.

**Table 2:** Multivariate analyses of clinical, pathological and virologic factors associated with poor prognosis in HCC patients.

Factors	Multivariate analyses	*P* value
	HR (95% CI)	
Age >50 years	1.031 (0.987–1.040)	0.336
Male	1.015 (0.421–2.900)	0.839
Cirrhosis	1.554 (0.652–3.707)	0.320
Tumor size >5 cm	1.602 (0.999–2.215)	0.050
Encapsulation	1.634 (0.731–2.201)	0.312
Multiple Tumor	0.523 (0.218–2.314)	0.287
CHILD Classification: B	0.616 (0.181–2.098)	0.439
TNM classification: II+III	1.342 (0.631–3.014)	0.330
AFP (ng/mL) >400	1.000 (1.000–2.030)	0.479
Portal vein thrombosis (+)	0.456 (0.104–2.002)	0.298
ALT > ULN	0.990 (0.976–1.003)	0.143
AST > ULN	1.015 (0.995–1.035)	0.145
Albumin < LLN	1.003 (0.954–1.054)	0.917
Bilirubin > ULN	0.983 (0.939–1.030)	0.478
Prothrombin time>ULN	1.022 (0.892–1.043)	0.093
PLT < 170	1.000 (0.995–1.004)	0.878
Alcohol use	0.809 (0.435–1.503)	0.502
HBV DNA > 6.0E+06 copies/ml	4.496 (1.218–15.116)	0.012
HBsAg > 500 IU/ml	1.887 (1.008–2.976)	0.057
HBV genotype C	1.268 (0.724–2.219)	0.406
TACO	3.007 (1.075–8.745)	0.029
High pAKT1 expression	4.022 (1.037–12.942)	0.039

Strong pAKT expression and TACO expression independently predict postoperative survival among HCC patients. A Cox regression analysis was used to identify factors associated with HCC prognosis, and the above factors were included in the analysis. Variables with *P* < 0.1 in a univariate analysis were further analysed using a multivariate Cox regression to identify the independent factors associated with poor prognosis. The data showed that a high level of pAKT (*P* = 0.039) and TACO expression (*P* = 0.029) were significantly associated with shorter overall survival in human HBV-related HCC.

**Table 3:** Correlation between pAKT and TACO mutations in HCC.

		HBV CP Status		
		TACO −	TACO +	
pAKT expression	Low, n(%)	23 (20.2%)	41 (36.0%)	*P* = 0.186
	High, n(%)	23 (20.2%)	27 (23.6%)	

The pAKT levels were similar in human HBV-related HCC samples with or without CP mutations in the HBV genome (*P* = 0.186). Categorical variables were analysed with a chi-square test. *P* < 0.05 was considered to indicate a significant difference.
