# Supplementary material for: HBV core promoter mutations and AKT upregulate S-phase kinase-associated protein 2 to promote postoperative hepatocellular carcinoma progression
Source: Sci Rep. 2016 Oct 25;6:35917. doi: 10.1038/srep35917 (PMC5078796; doi:10.1038/srep35917)
Supplement: Supplementary Information [file srep35917-s1.doc]

**HBV core promoter mutations and AKT upregulate S-phase kinase-associated protein 2 to promote postoperative hepatocellular carcinoma progression**

Lubiao Chen, Lin Gu, Yurong Gu, Hongbo Wang, Meihai Deng, Zania Stamataki, Ye Htun Oo, Yuehua Huang

**Table of contents**

Supplementary material & methods………………………………………………………2

HCC samples…………………………………………………………………………2

Western Blotting………………………………………………………………………2

Immunohistochemistry……………………………………………………………….2

Evaluation of Microvessel Density………………………………………………….3

Supplementary Figures.……………………………………………………………………4

Supplementary Figure S1……………………………………………………………4

Supplementary Figure S2……………………………………………………………5

Supplementary Tables…………………………………………………………….……… 6

Supplementary Table S1 ………………………………………………….………. 6

Supplementary Table S2 ……………………………………………….………… 7

Supplementary Table S3 ………………………………………………….……… 8

**Supplementary material & methods**

**HCC samples**

HCC diagnosis was made by one of the following methods: echo-guided liver biopsy, fine needle aspiration cytology, high AFP level (>200 ng/mL) plus at least one dynamic imaging study (dynamic computed tomography or magnetic resonance imaging, or one dynamic imaging study plus angiography (if AFP < 200 ng/mL). Tumors were completely resected, with a safety-margin of > 1cm. Postoperative follow-up was performed by ultrasonography, chest X-ray, AFP, and blood biochemistry every 1 to 3 months in the first year and every 3 to 6 months thereafter. Tissue samples were frozen to -80^o^C immediately, and serum in -30^o^C after surgical resection. HBsAg and HBeAg were measured by chemiluminescence assay (Roche e601, Roche Diagnostics).

**Western Blotting**

Cell lysates were separated by sodium dodecyl sulfate polyacrylamide gel electrophoresis (SDS-PAGE) and transferred onto nitrocellulose membranes. The membranes were incubated with primary antibody overnight at 4^o^C and subsequently probed with horseradish peroxidase (HRP)-conjugated secondary antibodies. The immunoreactive blots were visualized using an enhanced chemiluminescence regent (Pierce, Rockford, IL). Relative protein levels were quantified by densitometry analysis using NIH Image I (version 1.41).

**Immunohistochemistry**

Formalin-fixed and paraffin-embedded liver sections with a thickness of 4μm were deparaffinization in xylene and graded alcohols, hydrated and washed in phosphate buffered saline (PBS). For antigen retrieval, slides were microwaved in citrate buffer (pH 6) for 16 minutes. Endogenous peroxidase was inhibited by 3% hydrogen peroxide in methanol for 30 minutes, followed by avidin-biotin blocking using a biotin-blocking kit (DAKO, Corporation Carpnteria, CA). Sections were then incubated with anti-pAKT antibody (Cell Signaling) overnight in a moist chamber at 4°C, washed in Tris-buffered saline, and incubated in the corresponding secondary antibody at 37°C for 30 minutes. Slides were developed with DAB and counter-staining with hematoxylin.

**Evaluation of Microvessel Density**

To assess the microvessel density (MVD), the whole HCC collection was subjected to immunostaining with rabbit polyclonal anti-CD34 antibody. The liver tissues were screened at low power (40×) to identified the areas of highest MVD. Four highest MVD areas for each tumor were photographed at high power (200×), and the size of each area standardized using the Image 1.41 software. MVD was expressed as the percentage (mean ± SD) of the total CD34-stained spots per section area.

**Supplementary Figures and Legends**

**
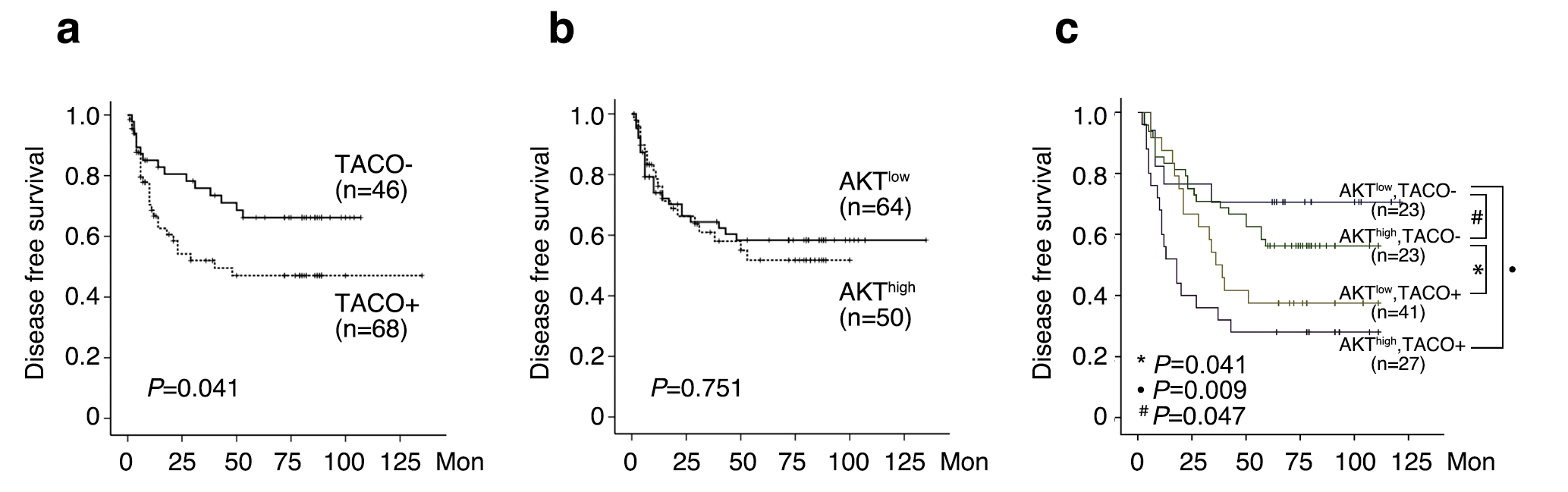
**

**Supplementary Figure S1. Association between postoperative survival and intrahepatic HBV CP mutations and pAKT levels.** (**a**) Comparison of disease-free survival between patients with and without TACO mutations. (**b**) Comparison of disease-free survival between patients with different levels of pAKT. (**c**) Combination of intrahepatic TACO mutations and pAKT as a predictor for disease-free survival.


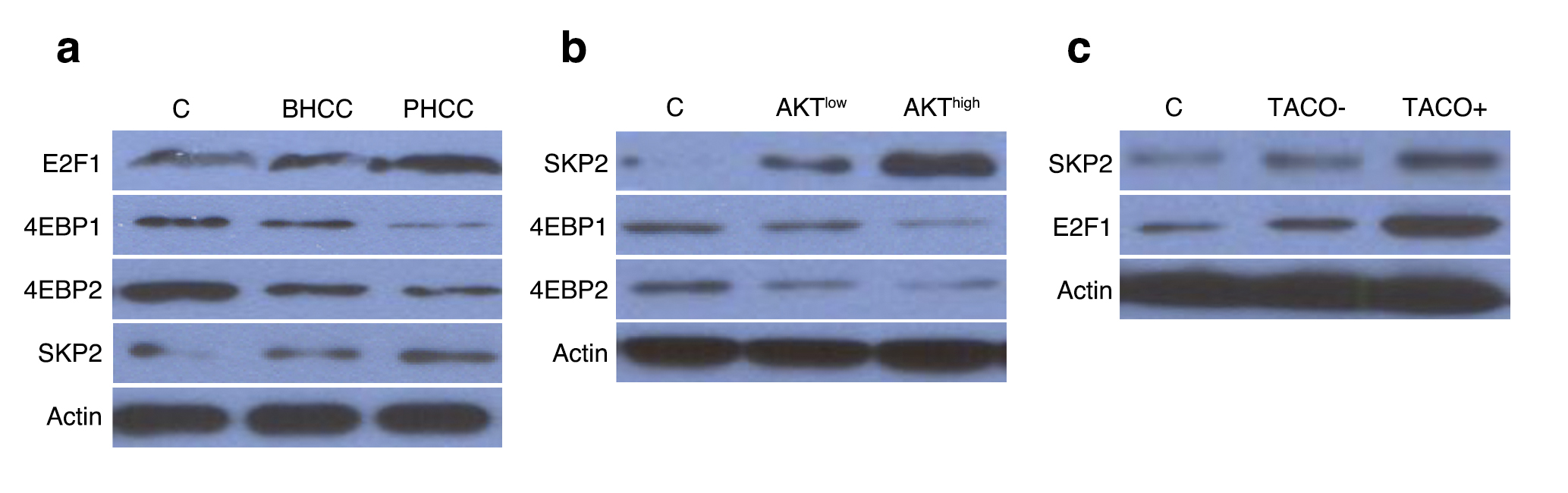


**Supplementary Figure S2. Up-regulations of SKP2 by AKT and TACO through independent pathways.** (a) Levels 4EBP1/2 and E2F1 in PHCC. (b) Decreased levels of translation inhibitor 4EBP1/2 in tumor tissue expressing strong pAKT. (c) Increased levels of transcription factor E2F1 in TACO HBV infected HCC.

**Supplementary Table S1**. **Clinical characteristics of patients included in the study.**

| Variables | Features |
| --- | --- |
| No. of patients | 114 |
| Age (y), mean±SD | 46.6±11 |
| Male, n (%) | 97 (85.1) |
| *Cirrhosis, n (%) | 83 (72.8) |
| Survival after partial liver resection (OS, mo), mean±SD | 46.6±18 |
| Disease free survival (DFS, mo), mean±SD | 23.5±10 |
| *Tumor quantity, n (%)  >=3  2  1 | 11 (9.6)  5 (4.4)  98 (86.0) |
| *Tumor size (cm), mean±SD | 7.43±4.5 |
| Encapsulation, n (%)  No  Yes | 40 (35.1%)  74 (64.9%) |
| AFP(ng/mL), mean±SD | 16025.8±1810 |
| ALT (U/L), mean±SD | 44.6±8.2 |
| *AST (U/L), mean±SD | 45.9±5.6 |
| *Albumin (g/L), mean±SD | 42.2±2.9 |
| Bilirubin (μmol/L), mean±SD | 16.7±4.8 |
| Creatinine (μmol/L), mean±SD | 81.5±10.2 |
| Alcohol use, n (%) | 42 (36.8) |
| Prothrombin time (second), mean±SD | 12.04±1.1 |
| TNM classification, n (%)  I  II+III | 68 (59.6)  46 (40.4) |
| CHILD classification, n (%)  A  B | 109 (95.6)  5 (4.4) |
| Portal vein thrombosis, n (%)  No  Yes | 109 (95.6)  5 (4.4) |

Of the 114 patients with HBV-related HCC after liver resection were included to analyze. Among above clinicopathological features, presence of cirrhosis, bigger tumor size, multiple lesions, higher AST, and lower albumin were factors significantly associated with patients of poor prognosis in current HCC cohort. Continued variables were compared by 2-sided Student *t* test. Categorical variables were analyzed with Chi-square test or Fisher’s exact test. * represents *P*<0.05, factors that significantly with shorter survival of <5 years. Abbreviations: AFP, alpha fetal protein; ALT, alanine aminotransferase; AST, aspartate aminotransferase.

**Supplementary Table S2. Multivariate Analysis of clinicopathological and virological parameters for disease-free survival in HBV associated HCC patients.**

| Parameters | No. of patients | DFS, month (Mean±SD) | Adjusted Hazard ratio  (95% CI) | *P* value |
| --- | --- | --- | --- | --- |
| Age (years)  >50  ≤50 | 43  71 | 62.4±12  46.1±15 | 1.245 (0.845-1.859) | 0.245 |
| Sex  Male  Female | 97  17 | 54.2±21  65.4±19 | 1.230 (0.614-2.859) | 0.369 |
| Cirrhosis,  Yes  No | 83  31 | 51.2±21  56.9±15 | 1.027(0.675-1.687) | 0.147 |
| Tumor size  >5cm  ≤5cm | 73  41 | 71.8±22  40.8±18 | 1.128(0.897-1.689) | 0.090 |
| Tumor quantity  ≥3  =2  =1 | 11  5  98 | 38.7±14  45.8±9.2  61.4±9.5 | 1.287(0.789-2.014) | 0.247 |
| Encapsulation  No  Yes | 40  74 | 40.9±12  61.6±13 | 1.125(0.610-1.741) | 0.147 |
| CHILD classification  B  A | 9  105 | 68.7±11  62.0±19 | 1.668(0.1000-2.658) | 0.080 |
| TNM classification  I  II+III | 55  59 | 60.1±15  46.5±21 | 0.784(0.567-1.258) | 0.258 |
| AFP (ng/ml)  >400  ≤400 | 47  67 | 68.7±25  45.8±10 | 1.978(1.231-3.247) | *0.041* |
| Ascites  No  Yes | 103  11 | 60.1±25  20.4±12 | 2.471(1.258-4.987) | *0.027* |
| Portal thrombosis  Positive  Negative | 5  109 | 48.7±21  69.7±18 | 1.182(1.026-1.400) | *0.050* |
| ALT (U/L),  >ULN  ≤ULN | 70  44 | 48.9±25  60.8±17 | 1.005(0.958-1.120) | 0.254 |
| AST (U/L)  >ULN  ≤ULN | 41  73 | 50.7±14  85.7±28 | 1.210(1.025-1.369) | *0.048* |
| Albumin  ≥40  <40 | 80  34 | 76.5±39  48.7±25 | 1.485(1.011-2.354) | 0.369 |
| Bilirubin (μmol/L)  ≥ULN  <ULN | 13  101 | 57.4±36  54.9±25 | 0.952(0.854-1.024) | 0.147 |
| Prothrombin time (sec)  ≥12  <12 | 12  102 | 42.1±30  76.8±17 | 1.784(1.057-3.175) | *0.041* |
| PLT (10E9/L)  <170  ≥170 | 41  73 | 56.8±36  51.2±29 | 0.828(0.412-1.690) | 0.781 |
| Alcohol use  Yes  No | 41  73 | 59.7±36  50.2±24 | 0.728(0.612-1.897) | 0.158 |
| HBV DNA (copies/ml)  >6.0E+06  ≤6.0E+06 | 33  81 | 36.7±14  71.0±19 | 1.638(1.024-2.687) | *0.018* |
| HBsAg (IU/ml)  ≤500  >500 | 60  54 | 56.8±28  62.8±32 | 1.668(0.987-2.587) | 0.368 |
| HBV genotypes  B  C | 66  48 | 69.7±30  41.6±28 | 1.589(0.657-1.847) | 0.367 |
| TACO status  Positive  Negative | 68  46 | 46.8±17  81.0±25 | 2.078(1.257-3.687) | *0.010* |
| pAKT1 expression  High  Low | 50  64 | 43.5±15  63.6±31 | 1.334(0.824-2.161) | 0.080 |

**Supplementary Table S3. List of the primary antibodies used.**

| Protein | Antibody | Company |
| --- | --- | --- |
| Ki67 | Rabbit monoclonal | ThermoFisher, USA |
| AKT | Rabbit polyclonal | Cell Signaling Technology, Beverly, MA |
| pAKT1 | Rabbit monoclonal (ser437) | Cell Signaling Technology, Beverly, MA |
| Cyclin D1 | Rabbit polyclonal | Santa Cruz Biotechnology, Santa Cruz, CA |
| Cyclin E | Rabbit polyclonal | Santa Cruz Biotechnology, Santa Cruz, CA |
| p21 | Mouse monoclonal | Santa Cruz Biotechnology, Santa Cruz, CA |
| p27 | Mouse monoclonal | BD Bioscience, Franklin Lakes, NJ |
| SKP2 | Rabbit monoclonal | Abcam, UK |
| 4EBP1/2 | Rabbit monoclonal | Cell Signaling Technology, Beverly, MA |
| β-Actin | Rabbit polyclonal | Santa Cruz Biotechnology, Santa Cruz, CA |
| E2F1 | Mouse monoclonal | Cell Signaling Technology, Beverly, MA |
| CD-34 | Rabbit polyclonal | Abcam, UK |
